# Acceptability and feasibility of the World Health Organization's Caregiver Skills Training Programme (WHO CST) delivered *via* eLearning, videoconferencing, and in-person hybrid modalities in Hong Kong

**DOI:** 10.3389/fpsyt.2022.915263

**Published:** 2022-09-07

**Authors:** Janet Siu-Ping Lau, Simon Man-Kin Lai, Florence To-Sau Ip, Paul Wai-Ching Wong, WHO CST Team, Chiara Servili, Erica Salomone, Laura Pacione, Stephanie Shire, Felicity L. Brown

**Affiliations:** ^1^The University of Hong Kong, Hong Kong, Hong Kong SAR, China; ^2^WHO CST Regional Technical Focal Point, Geneva, Switzerland; ^3^Department of Mental Health and Substance Use, World Health Organization, Geneva, Switzerland; ^4^The University of Milano-Bicocca, Milan, Italy; ^5^Division of Child and Youth Mental Health, Department of Psychiatry, University of Toronto, Toronto, ON, Canada; ^6^The University of Oregon, Eugene, OR, United States; ^7^Research and Development Department, War Child Holland, Amsterdam, Netherlands; ^8^Amsterdam Institute of Social Science Research, University of Amsterdam, Amsterdam, Netherlands

**Keywords:** World Health Organization's Caregiver Skills Training Programme, WHO CST, eLearning, videoconferencing, in-person, developmental delays or disabilities, parenting, COVID-19

## Abstract

**Background:**

Local children with developmental disabilities were deprived of learning opportunities due to recent social and health incidents, resulting in elevating challenging behaviors and familial conflicts. This study explored the acceptability and feasibility of the World Health Organization's Caregiver Skills Training Programme (WHO CST) in alternative delivery modes under new normal and post COVID-19 period.

**Method:**

CST was delivered *via* eLearning (EL), videoconferencing (VC), and in-person hybrid (IP) modes to 34 parent-child dyads, being randomly assigned to modes of asynchronous non-interfering EL (*n* = 9), synchronous with online coaching VC (*n* = 7), synchronous with in-person coaching IP (*n* = 9) and Wait-list Control WLC (*n* = 9). Data from two standardized scales of General Health Questionnaire (GHQ-12) and Strengths and Difficulties Questionnaire (SDQ), and Post-session and Home Visit Feedback Form by Caregivers that included both structured and open-ended questions were collected before and after intervention. Both quantitative and qualitative approaches were used in studying the collected data.

**Results:**

High levels of acceptability and feasibility of the training programme were supported by ratings on comprehensiveness and relevance, agreement with their personal values, duration, and usefulness. IP and VC groups yielded more positive changes than EL and WLC groups with 3, 16, 13, and −3% in General Health Questionnaire (GHQ-12), −13, −15, −6 and 0% in Difficulties-total, and 36.5, 35.5, 5.8 and 2.4% in Prosocial Scale at Strengths and Difficulties Questionnaire (SDQ) for EL, VC, IP, and WLC groups respectively from baseline to 12 weeks after intervention. Results from two standardized scales echoed with qualitative observations that the programme helped improve caregivers' well-being, child's communication, and behaviors across intervention groups.

**Conclusions:**

Current findings revealed that CST delivered in three alternative modes were acceptable and feasible, and yielded positive impacts toward both caregivers and children. In-person coaching, and skill-practicing sessions were effective in mitigating child's challenging behaviors while personal interaction, either face-to-face or virtual, is a significant factor in uplifting caregivers' well-being, whereas the self-learning model was appreciated by the busy caregivers. In clinical practice, needs and goals of families and the constraints of remote interventions at the settings should be balanced.

## Introduction

Twenty-four months under restricted movement and social interactions, people in Hong Kong have been exploring new ways of living to restore normal life. However, twenty-four months in early childhood is more than one-third of the “golden development period” (1) which comprises more than 80% of a person's brain development (2, 3). During this critical fraction of development, children can learn and experience rapid development in speech, motor, cognition, and emotions (1, 4) while others require significant support to mitigate their learning needs. Unfortunately, most of the people in Hong Kong have been experiencing an “unprecedentedly stretched lockdown” for over 2 years. The social upheaval due to large protests in June 2019 led to school suspension as transportation was handicapped with potential dangers on the street for more than 6 months. This was followed by social distancing rules that were enacted in Hong Kong due to the pandemic in January of the following year. Therefore, children were kept confined at home and received online schooling for another six months. Lockdown is, indeed, meant to protect the vulnerable, however, for children with developmental disorders or delays the loss of face-to-face schooling and adequate services leads to a dearth of learning opportunities, and oftentimes, elevated unresolved challenging behaviors (5), as well as familial conflicts, including child abuse and domestic violence within the households (6–8). In view of this service gap and elevated parenting needs during this challenging time, this study was designed to examine the acceptability and feasibility of a worldwide, evidence-based parenting programme, the World Health Organization's Caregiver Skills Training Programme (WHO CST or CST), delivered *via* eLearning (EL), videoconferencing (VC), and in-person hybrid (IP) modes in Hong Kong. This study examines alternative delivery practices leveraging remote delivery modalities to provide access to services under challenging conditions to help these parents stay positive and make use of their time for teaching their children at home under the new normal.

## Background

“Developmental disabilities” (DD) is a comprehensive term to describe the lifelong chronic neurodevelopmental conditions of early childhood that include but not limited to disorders of intellectual development and autism spectrum disorder (ASD), which share common features: discrepancies in physical, cognitive, linguistic, social, and adaptive functioning from the normative developmental milestones (9).

It is estimated that 65 out of 10,000 people have autism globally (10). In the United States, 1 in 44 of the 8-year-old population was diagnosed with ASD in 2018 (11). In Hong Kong, approximately 9.5% of the population with ASD reported various levels of difficulties (12) and the newly diagnosed cases have increased 3-fold, from 755 in 2006, to 2,021 in 2015 (13). On top of the queuing time for diagnosis, the long waiting time ranging from 12 to 18 months (14) for special educational support puts caregivers in a stressful situation as support is minimal in meeting the children's and their own needs (15).

Parenting can become more stressful for parents of children with ASD. Children with developmental delays or disabilities may need additional support to acquire daily living skills and communicate with others (16). Parents can benefit from services designed to help caregivers learn strategies to support their children with special needs. However, engaging in these services can add burden (17) on top of a heavy load of daily chores (18) as well as the challenges arising from the affected family functioning and their personal well-being (16).

With the restrictions of the social movements followed by the onset of COVID-19 in June 2019, children in Hong Kong have been unprecedentedly locked down. Due to the lockdown, children with developmental delays/disabilities could not access intervention because servicing centers were closed. The shutdown was meant to protect these vulnerable children. Nevertheless, staying at home, having online classes with plenty of distractions, and being unable to access adequate services did not only limit learning opportunities and energy outlets for children with higher needs, but led to the elevation of children's challenging behaviors and familial conflicts within the households. While the psychological impacts of being locked down were yet to be reported, the burden of parenting children with special needs was heightened with the sudden cancellation of school, unprepared daily nurturing roles, and confining children with special needs in an average living space of 13.5 square meters per person (19).

On the other hand, the pandemic also impacted the lives of working caregivers. Due to the lockdown situation, flexibility became even more essential as parents were spending time taking care of the stay-at-home school-aged children in the daytime while also working from home. With more time nurturing their children on their own than before, these caregivers experienced a sense of helplessness or inadequacy in their parenting (5). Wong and his colleagues (5) also found that caregivers indicated the need for a programme that could be more flexible in terms of time and place, to fit the office schedules of working parents. In addition, some practitioners revealed the challenges of having face-to-face home visits under the health and safety guidelines and regulations of their affiliated organizations. Thus, to address all these challenges, these voices called for alternative delivery modes of parenting programmes in Hong Kong, especially in this critical period.

There is growing evidence that caregiver-mediated programmes can empower caregivers to support children's communication and engagement (20), and help reduce challenging behaviors (21, 22), in turn, leading to better developmental, behavioral, and family functioning outcomes (21, 22). Furthermore, caregivers' stress can be alleviated by relevant interventions by encouraging caregivers to reach out for professional and informal support (23–25).

Following advocacy of high-quality development for children under the WHO Global Strategy for Women's, Children's and Adolescents' Health (26) and the growing needs of families of children with developmental disabilities (27), the World Health Organization (WHO) developed the Caregiver Skills Training Programme for Families of Children with Developmental Delays and Disabilities (CST). The CST programme is based on a common elements approach and informed by the findings of numerous meta-analyses (21, 22, 28) and was developed in consultation with experts and parents' associations from all WHO regions with financial support from Autism Speaks (29). The programme is a scalable, affordable parenting programme which is applicable to children with developmental delays and disorders in low and high resources settings by non-specialists (30). The programme targets caregivers of children aged 2 to 9 years with a developmental delay or disability, especially in the domains of social interaction and communication, although a diagnosis is not required. The aim is to teach caregivers basic strategies to promote their child's development and adaptive behaviors, to improve daily interactions between caregiver and child, as well as support caregivers' self-care (31). Under the framework of implementation science, CST is currently in the stage of pilot-testing in more than 30 countries (31) and several randomized controlled trials are underway and more evidence-based research is undergoing publication. Overall, the programme has shown excellent acceptability and feasibility in both high- and low-resource settings (32, 35) and indicators of clinical effectiveness (33, 34).

### Different delivery modes

Financial constraints, commuting difficulties, lack of childcare, geographical distance, long waiting times, and time commitments are regarded as major barriers to conventional services (36–38). Although telehealth programmes provide a solution to the traditional servicing mode, the utilization of technology in the health-related field remained stagnant due to skepticism about the potential risks and unforeseen effects of their applications (39). However, the outbreak of COVID-19 has become the turning point, driving the field to give up conventional therapeutic approaches and embrace the potential benefits of delivering services through technologies (39–43).

The enactment of social distancing measures facilitates the escalating application of technology among mental health service providers, resulting in a rapid shift from in-person mode to telehealth services (43). Previous meta-analyses had shown that tele-mental health care could generally provide effective and adaptable solutions for the care of mental issues (44). Nevertheless, professionals also reported challenges in different areas, including technical problems (45–47), privacy protection concerns (48), and the need to adapt to an unfamiliar interaction pattern (5, 49) to foster a facilitative environment and supportive relationships with clients (41).

#### Delivery of social services using technology

While the efficacy of applying new technologies in social services is under investigation, there have been many telehealth interventions established in the community (50). The use of modern technology can accelerate different telehealth services in different manners, such as personal tele-psychotherapy, self-directed learning, and online group workshops, including those with high complexity and long duration, which can be supplemented by different delivery modalities and instructional design calibrated to meet the needs of the target clientele (51) such as eLearning, videoconferencing, and conventional in-person modes.

##### eLearning

ELearning can be regarded as the dissemination of knowledge through a technology-based learning system where learners can acquire knowledge at their own pace. A meta-analysis on the effectiveness of Internet-based eLearning across different beneficiaries suggested that eLearning was at least as effective as conventional learning methods (52). Some evidence illustrated that healthcare professional behaviors were improved better than without guidance at all (51, 53). Many studies on the adaptation of in-person training or therapies for online delivery revealed positive results in knowledge attainment, stress, and fidelity to treatment plan (54–58).

Evidence-based ASD interventions have also been adapted for delivery using technology. One three-month intervention programme for children with autism delivered by an app in Australia revealed that improvements in language and social communication skills were not only found in the posttest but prolonged at 12 months post-intervention assessment (59). Wainer and Ingersoll piloted a similar web-based self-directed telehealth programme, resulting in encouraging results that participants were able to learn about reciprocal imitation training (RIT) and increase the application of RIT techniques afterwards (57). Another Self-Directed Learning Programme showed significant differences between the treatment group and the control group on all dependent measures, including implementation fidelity on Pivotal Response Training (PRT) procedures, language opportunities, functional verbal utterances, and observed parent confidence (56).

Nevertheless, the findings of these studies indicated that participants suggest adding interactive remote coaching sessions to a self-directed eLearning parenting programme, which can be crucial and essential for online parent training programmes, including weekly telephone calls or email correspondence, peer groups, and regularly personalized coaching sessions (60). Given the limited but essential need for interaction with others, research has begun to explore how to use videoconferencing to facilitate remote feedback and support for parents of children with ASD (53, 55, 61–64).

##### Videoconferencing

Internet-based videoconferencing technology offers a structure like face-to-face meetings, providing real-time opportunities for therapists and patients to interact from different geographic locations. Videoconferencing can serve as a sole intervention medium or a supplementary component enhancing participation and interactions between therapists and participants. Studies revealed that parents find the adoption of virtual psychotherapy or training acceptable, easy to use, and effective (49, 58, 63, 65). Several empirical studies showed that Internet-based teaching or a combination of multiple strategies can provide promising results in applied-behavior-analysis (ABA) intervention (53, 55, 63, 64, 66–68).

Videoconferencing intervention, adding to self-directed programmes can be a solid combination for the programmes that last for several weeks or months. One combined-mode programme working with families of ASD children revealed encouraging outcomes in implementing behavioral management skills for both daily and play activities (69). Another study on combined modes, including an additional 13 hours of instructional guidance and 4 hours of group coaching for children with autism showed a significant improvement in intervention techniques (70), which shows that expert guidance and support, even virtual, can be helpful to achieve implementation fidelity.

In view of skills attaining programmes *via* online means, research shows that these programmes provide promising results for both parents and their children with ASD in different aspects. A three-week online training programme on a weekly basis with instructional content, group discussions, and problem-solving support is sufficient to demonstrate behavioral improvement in autistic children and reduction in parenting stress (53). Fisher and colleagues (55) (2020) conducted a comprehensive pre-post study on teaching applied-behavior-analysis skills to parents of children with ASD to evaluate the differences between the treatment group and the control group at individual and group levels, under structured and play contexts which demonstrated a significant improvement in the treatment group for the measures at posttest. These two studies on the efficacy of applying technologies to parenting programmes for ASD or DD families showed positive outcomes; however, comparing the outcomes with those through the conventional face-to-face programme or self-directed mode is needed.

#### Comparison of service delivery modalities: In-person and videoconferencing modes

There is a paucity of studies comparing the effectiveness of interventions facilitated by videoconferencing with those delivered in-person conventionally. Therefore, it is difficult to conclude whether videoconferencing or in-person delivery is more effective. Some studies show that videoconferencing interventions are as effective as in-person interventions. For instance, a qualitative study aimed at improving parental mental health with a mixed mode of three face-to-face sessions and five videoconferencing sessions was conducted to explore the acceptability and feasibility of this mixed service mode. Results indicated that parents valued videoconferencing as an acceptable delivery means of intervention programme while videoconferencing sessions promoted a higher attendance (49). In Luxton and colleagues' study (71), participants were allocated to either conventional in-person settings or home-based videoconferencing modes. Results suggested that participants in both groups showed reductions in hopelessness and depressive symptoms; however, there was no statistical significance between the groups. These suggest the effectiveness comparison of the two delivery modes is inconclusive.

#### The current study

As highlighted by Duan and Zhu (72), sufficiently dynamic and flexible psychotherapeutic interventions should be adapted quickly to different stages of the pandemic. Emphasis on flexibility and adaptability is a common theme of research in this special issue (42). Indeed, CST was developed to be adapted to the cultural, socioeconomic, geographic, and resource context in which it is used (30). From 2018 onwards, the University of Hong Kong has adopted and implemented CST in Hong Kong. It was thereafter tested in two phases: pre-pilot and pilot tests. The current study follows the effectiveness studies on the adapted CST materials of version 1.04 being conventionally delivered while further studying different feasible remote intervention modes in Hong Kong. In view of the aforementioned service gap and elevated parenting needs during this challenging time, this study aims to examine the acceptability and feasibility of an evidence-based parenting programme, the World Health Organization's Caregiver Skills Training Programme (WHO CST), to be delivered by eLearning (EL), videoconferencing (VC), and in-person hybrid (IP) modes of delivery in Hong Kong. The programme supports caregivers' skill attainment to strengthen children's communication and joint engagement while reducing challenging behavior. Together, these skills support children's adaptive behaviors and family functioning that ultimately lead to an improved caregiver-child interaction. The programme also helps strengthen caregivers' coping skills, psychological well-being, and quality of life, as well as mitigate the stigma against developmental disorders (30).

## Methodology

### Participants and randomization

Participants were recruited through a convenience sampling method by sending recruitment messages *via* the university bulk email services and *via* social media to the caregivers of children suspected of having developmental needs. They gave their consent for research participation and sharing of their demographic data along with an initial set of screening questions based on the inclusion and exclusion criteria, which was done *via* the encrypted platform along with an enquiry hotline supported by a research assistant. Participants were: (a) primary caregivers residing with a target child in Hong Kong, (b) able to communicate in Cantonese, and (c) literate in Chinese. The target children were: (a) between 2 and 6 years old, (b) with symptoms of autism spectrum disorder or other developmental disorders or delays, (c) with a score of 3 or above on the Modified Checklist for Autism in Toddlers (M-CHAT). The randomization was conducted using Microsoft Excel to allocate participants to eLearning (EL), in-person hybrid (IP), videoconferencing (VC), and wait-list control (WLC) groups, with 17, 18, 17, 18 participants respectively. This study was approved by The Human Research Ethics Committee of Hong Kong University (HKU) (EA200178) and all data collection was performed in accordance with HKU guidelines and regulations.

### Settings and materials

The present study took place in both the virtual online setting and HKU campus. A standard Caregiver Child Interaction (CCI) toy kit was given to each participating family upon admission as a standardized tool to record the caregiver-child interaction for the three-time points. The CCI kit included wooden blocks and plastic balls, nesting cups, toy vegetables and fruits with velcro, a non-assembled stove with kitchen utensils, figurines, and a drawing set, which covers a developmental range of play from the earliest simple play skills (e.g., roll a ball, take apart Velcro fruit) through symbolic level pretend play (e.g., pretending to be a chef, pretending a nesting cup is an airplane). Also, Participant Booklets were sent to the IP and VC participants only.

### The intervention

The intervention followed the CST Facilitator Guide version 1.00 (74, 75) and the WHO Parent Skills Training Programme for Caregivers of Children with Developmental Disorders: Monitoring and Evaluation Framework provided by the World Health Organization (75). The intervention is an originally evidence-based programme package based on the principles derived from behavioral and social learning approaches, child development theories as well as positive parenting approaches focusing on training (coaching) parents or caregivers to play and home activities as daily routines and opportunities for learning and development (32, 33). It also emphasizes a task-shifting approach, in which paraprofessionals or non-specialists can be trained to deliver the programme to parents or caregivers to support their child's engagement in daily home and play activities. It also emphasizes a transdiagnostic approach such that children do not need to meet the diagnostic criteria for ASD or other pervasive developmental disabilities in order to access the programme. The programme consists of nine group sessions and three individual home visits. The content of the sessions includes (a) promoting joint engagement (sessions 1a, 1b, and 2), (b) promoting communication (sessions 3–4), (c) promoting positive child behavior and managing challenging behaviors (sessions 5–6), (d) learning new skills (session 7), and (e) empowering participants' troubleshooting and self-care capability (session 8). The programme strategies were explained through illustrated stories, role-plays, video demonstrations, group discussions, and guidance for home practice. The skills were further strengthened with the three home visits which were held before the first group session, after session four, and after the last group session.

The programme materials were adapted and translated from English to Chinese and back-translated following the CST Draft Adaptation and Implementation Guide Field Version 1.01. The adapted content was revised and endorsed through three adaptation meetings with various stakeholders, community experts, service providers, and caregivers, and the feedback from the need assessment study (5). Minor adaptations included agreements on the name of the programme and the proposed terms in Traditional Chinese (e.g., Joint Engagement), localized daily examples (e.g., irrelevant cause to developmental delays like “Sins of Family” was not emphasized in Hong Kong cultures and was reordered on the list), and additional support to participants to join the sessions (e.g., babysitting services).

With the programme materials remained constant, only the delivery modalities varied in this study. The three experimental groups were eLearning (EL), videoconferencing (VC), and in-person hybrid (IP) modes varying with the level of remote interventions and synchronicity. Detailed adaptations for each delivery mode are described in Appendix.

#### eLearning

This was an asynchronous, non-interfering mode with the programme materials including the digital versions of CST participant booklets and pre-recorded videos demonstrating CST skills and strategies provided through an encrypted online platform. This was to highlight the self-learning nature that the caregivers could learn CST skills online within the assigned weeks at their own pace, and free from location and time restrictions. Participants were given a unique credential to access the programme materials on a weekly basis and to submit their answers of the post-session quizzes and feedback forms after each session *via* the platform. The administrator would send out gentle reminders to the participants who had not sent in the answers and feedback on the fifth day of the assigned week. Data collected were encrypted and could be accessed by restricted researchers. The programme schedule together with the submission of the caregiver-child interaction videos over the standard CCI kit followed that of the other two experimental groups. No practice in pairs and no coaching was involved while wellness exercises and live demonstrations were replaced by pre-recorded demonstration videos.

#### Videoconferencing

This was a completely online mode where all home visits and weekly group sessions were conducted by a pair of qualified facilitators *via* Zoom videoconferencing software. Participants interacted with other parents with similar parenting experiences online despite their location. They received the Participant Booklet and the standard CCI kit by post before the intervention. Wellness exercises and live demonstrations were played by pre-recorded videos. Sharing and plans for home practices were discussed in the sessions and facilitators lectured on the programme key message and skills review. All components of the home visits were conducted online, including the observation of the parent-child interaction, facilitator verbal coaching, and goal setting.

#### In-person hybrid

The in-person delivery mode was originally planned as face-to-face group sessions at the HKU campus and individual home visits at the participants' homes. However, following the outbreak of COVID-19, a hybrid mode was employed to meet social distancing measures while the personal skills drilling components could be preserved. The changes included turning the nine face-to-face group sessions into online sessions with Participant Booklet received in advance while the three home visits remained in-person sessions with an addition of three 1-hour live skills practice sessions at the third, sixth and ninth sessions. In this way, experiential learning of the skills and peer support was made possible. Like VC, the didactic sessions were conducted online by a pair of qualified facilitators weekly at a fixed time slot with wellness exercises and live demonstrations through playing pre-recorded videos. Sharing on and plans for home practices were discussed in the group while facilitators lectured on the programme with key message and skills review. All the home visits were conserved as the original CST home visits and conducted in person, where parent-child interaction, facilitator coaching, and goal setting were made interactive and done face-to-face with the two facilitators (75).

### Master trainers and facilitators

Two years prior to this study, a pre-pilot study and a pilot study were conducted in Hong Kong to test out the Hong Kong-adapted CST materials and the knowledge transfer from the CST trainers to master trainers in Hong Kong, and further to the facilitator level. Eight master trainers were trained by CST trainers in Hong Kong in early 2019. This study was conducted by the principal master trainer who had achieved over 98% at the implementation fidelity assessment. The principal master trainer engaged in the programme adaptation and implementation process, conducted several CST groups at the pre-pilot stage, as well as trained and supervised the facilitators at the pilot stage.

Four students attaining a master's degree in Social Science at the University of Hong Kong enrolled in the standardized 7-day CST facilitator training and were thereafter supervised by the in-charge Master Trainer, including didactic theories, role-play, demonstrations, and live practices. Following two months of supervised practice, the facilitators' fidelity of CST intervention strategy implementation was assessed. Each facilitator recorded a 10-min interaction with a child with a disability while applying the strategies in the context of play and home routines. All facilitators passed the 11-item Adult/Child Interaction Fidelity Rating of version 1.04 at 75% before the intervention commenced. Furthermore, the facilitators' group facilitation skills were evaluated through a live session demonstration with their peers engaged as caregiver participants. The intervention was conducted in pairs and continual supervision was secured throughout the programme. Group facilitation skills were evaluated using the 22-item ENhancing Assessment of Common Therapeutic Factors (ENACT) (73) during the intervention.

### Assessments

#### Baseline measures

At baseline, participants' socio-demographic information along with a screening measure, the Modified Checklist for Autism in Toddlers (M-CHAT) (76), and a set of baseline measures, including the Caregivers Skill and Knowledge questionnaire, 12-item General Health Questionnaire (GHQ-12) (77), and 25-item Strengths and Difficulties Questionnaire (SDQ) (78) were collected.

#### Fidelity measures

Each group was run by a pair of facilitators who were assessed and supervised by a qualified Master Trainer. Before the programme, the facilitators had undergone an intensive 7-day Training of Trainer (CST ToT), and subsequently were qualified as facilitators by achieving at or over the fidelity of 75% using the 11-item Adult/Child Interaction Fidelity Rating of version 1.04, and over 2.5 (out of four) at the score of 22-item ENhancing Assessment of Common Therapeutic Factors (ENACT) (73) after 4 months of supervised practice.

#### Evaluation of acceptability

The acceptability of the programme in various implementation modes was evaluated by the feedback on the comprehensiveness and relevance, value conflict, duration of each session and on perceived usefulness of home visits and live coaching after each home visit by caregivers, and the post-session feedback concerning perceived acceptability of the programme, including contents of the sessions, level of engagement, acceptance of the caregivers, and level of involvement of caregivers by facilitators.

##### Post-session and Home Visit Feedback Form by Caregivers

Post-session Feedback Form by Caregiver and Home Visit Caregiver Feedback Form were completed by the caregivers after each CST group session and after each home visit accordingly. Six questions in the Post-session (PS) Feedback Form by Caregivers and three questions in Home Visit (HV) Caregiver Feedback Form were especially adopted to assess the acceptability of the programme being conducted in different modes.

Responses over the Post-session Feedback by Caregiver were selected to assess the comprehensiveness and relevance of each session. Comments of two other questions were evaluated to study the agreement with participants' and their family members' personal values. The appropriateness of time used in each group session and each home visit were evaluated. Lastly, the perceived usefulness of home visits and live coaching comments were asked in the Home Visit Feedback Form by Caregivers.

##### Facilitator's Feedback Form

Facilitator's Feedback Form concerning the perceived acceptability of the programme, including contents of the sessions, level of engagement, acceptance of the caregivers, and level of involvement of caregivers of each group session were collected after each session.

#### Evaluation of feasibility

Feasibility of CST conducted through different delivery modes were assessed in terms of participants' completion rate, attendance, adherence to home practice, observer's feedback on the involvement of the participants and implementation of the group sessions. These aspects were included in the following measures.

##### Caregiver diary

Caregivers' adherence to home practice was collected after home visit 2 (T1) and home visit 3 (T2). Caregivers reported their practice frequency on a weekly basis and the amount of time they practiced the “Skills and Strategies” each day.

##### Observer's Feedback Form

Group sessions' intervention fidelity was reported by observers after each group session, who was the facilitator not in-charge of the teaching that session, in rating the participants' degree of comfort, enthusiasm/interest, and level of involvement in planning home practice.

##### Facilitator's Feedback Form

Complexity, amount of the sessions' contents, and perceived preparation for the sessions were rated by the teaching facilitator to evaluate the implementation of the programme after each group session.

#### Clinical outcome measures

##### Quality of Life as parent outcome

The General Health Questionnaire (GHQ-12) is a 12 self-assessed instrument to reflect the mental wellness of respondents on a 4-point Likert-type scale from 0 (not at all) to 3 (always). Confirmatory factor analyses (CFA) study (77) showed that the general factor was strongly associated with symptoms of insomnia and mental health. The rating of the 12 questions (with those of questions 1, 3, 4, 7, 8, and 12 being reversed) were summed up to get the total score with a cut-off score of 12. The lower the score, the better the wellness. Participants were asked to complete the form at baseline (T0), after home visit 2 (T1), and after home visit 3 (T2) to investigate if there were changes in the caregivers' perception of their qualities of life along with the intervention, and to compare if there were any changes between learning modes.

##### Strengths and Difficulties Questionnaire as child outcome

The Strengths and Difficulties Questionnaire (SDQ) is designed to score children, aged 3 to 16, on their behaviors. Confirmatory factor analysis (79) demonstrated strong evidence for convergent and discriminant validity. There are 25 items in SDQ classified into 5 scales including one strength scale as prosocial skill and four difficulty scales in conduct problem, hyperactivity, emotional problem, and peer problem. These four scales are added together to generate a total difficulty score. Each scale consists of 5 items with a 3-point Likert-type scale, with “somewhat true” always scored as 1, but the scoring of “not true” and “certainly true” varies with the item scored as 2 or 0. The lower the score represents the better the condition. Participants were asked to complete the form at baseline (T0), after home visit 2 (T1), and after home visit 3 (T2).

#### Qualitative measures

##### Open-ended questions from Post-session and Home Visit Feedback Form by Caregivers

Comments from three open-ended questions in Post-session (after each group session) and Home Visit Feedback Form (after each home visit) by Caregivers were triangulated and supplemented with the quantitative data in this study. The questions were on the suggestions to improve each session, improvements to each home visit, and video-recording arrangement of each home visit. The feedback was investigated in detail and outstanding perspectives were reported.

#### Data analysis

This study employed the mixed-methods approach. Quantitative data were collected through questionnaires from CST protocols (75) and clinical outcome measures while qualitative data from the open-ended comments were gathered to corroborate and triangulate findings.

##### Quantitative data analysis

Data were input into SPSS 25 for cleaning, managing, and analysis. Central tendency and variance were used to characterize the general trend of the data. Descriptive analyses were conducted on all measures. In view of the small sample size, descriptive statistical analyses would be adequate to reflect the reality of implementing the CST in Hong Kong settings. Two-way ANOVAs were conducted to test if there were differences across the four conditions and between the T0 and T2 for GHQ-12 and SDQ.

##### Qualitative data analysis

A thematic analysis (80, 81) was done on qualitative data collected from each session and each home visit given by all attending participants across the three intervention groups while wait list control groups only gave comments on each home visit. The comments were recorded and collected in written form from the encrypted platform. The data were sent to two coders to read and generate the codes independently before they came to a consensus to identify a final set of codes. They continued to review the code set and their theme and sub-theme classification before the actual coding process on all the comments. The participants were coded with a group identifier as EL01, EL02, IP01, IP02, VC01, VC02, WLC01, WLC02 (...) to uphold privacy and confidentiality.

## Results

### Participants

**Figure 1:**
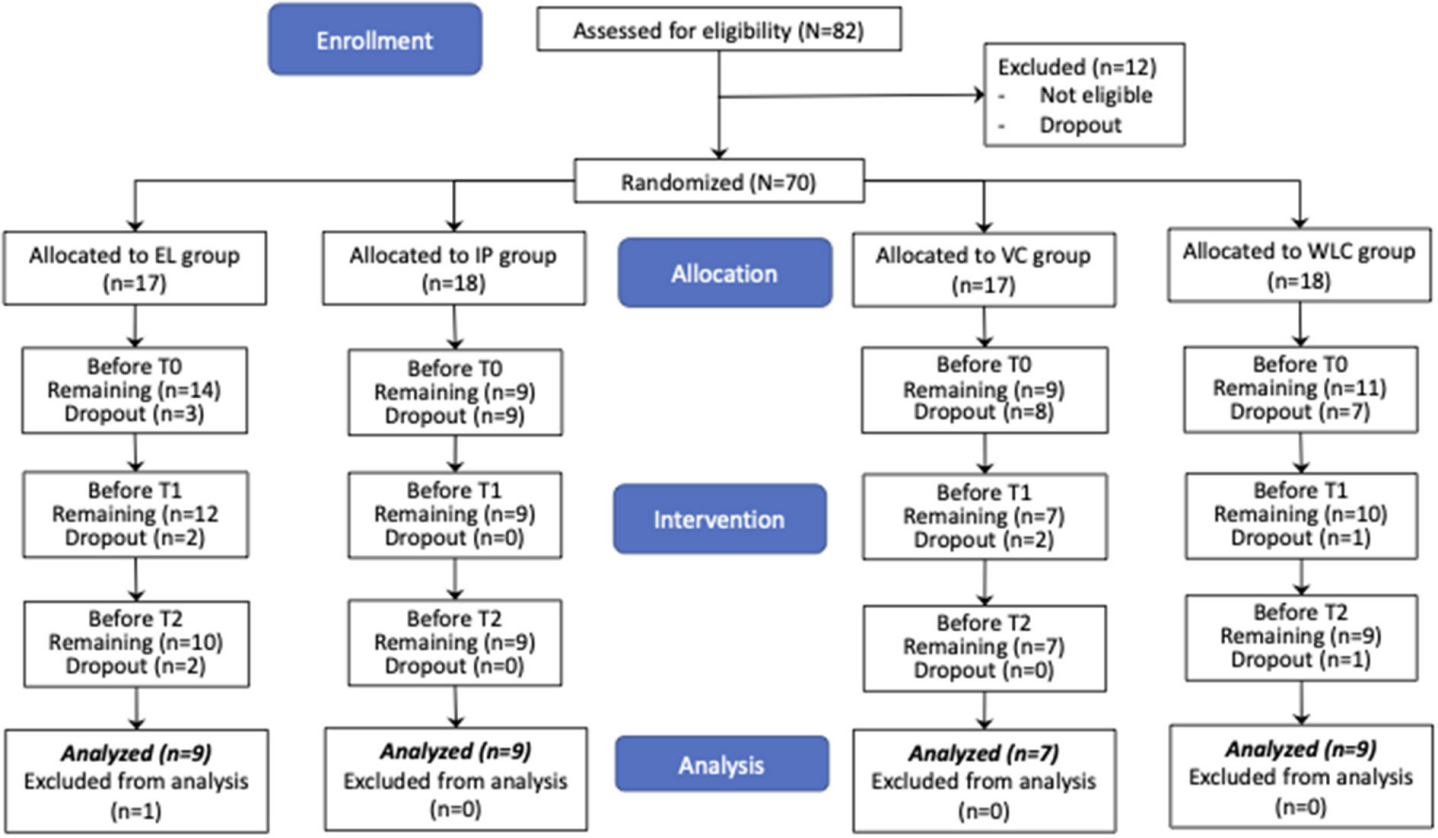
CONSORT 2010 flow chart for participant recruitment & processing.

Referring to Figure 1, a total of 82 participants showed interest in the programme, and 70 caregivers who fulfilled the criteria stated in 2.1.1 were considered eligible for the study. The participants were randomized through excel functions and assigned into eLearning (EL), in-person hybrid (IP), videoconferencing (VC), and wait-list control (WLC) groups, with 17, 18, 17, 18 participants, respectively. They gave the Informed Consent Form. Three EL, nine IP participants, eight VC, and seven WLC participants withdrew before any intervention. A total of 8 participants (EL = 4, VC = 2, WLC = 2) withdrew from intervention due to the worsening pandemic situation. By T2, a total of 34 participants across EL (*n* = 9), IP (*n* = 9), VC (*n* = 7), and WLC (*n* = 9) participants completed the programme.

Eighty-eight percent (30 out of 34) of the caregivers were female, and their average age was 39 years (SD = 3.52; range = 32–47). Eighty-eight percent of them were born in Hong Kong, and the rest were born in Mainland China. All completed secondary school or above, of which sixty-two percent had completed tertiary education. Over 60% of the children had more than one caregiver. Sixty-eight percent of the participants had a full-time job, one of the 4 part-time parents worked at home, while others did not go to work. Eighty-two percent (28 out of 34) of the target children were male, and the average age of the children was 4.4 years (SD = 1.4; range = 2–7). All of them were born in Hong Kong. The demographic characteristics of the participants of all groups were summarized in Table 1. Except for “who is the main caregiver,” there are no significant differences in other demographic characteristics between groups.

**Table 1:** Demographic characteristics of the participants.

	**EL (*n =* 9)**	**IP (*n =* 9)**	**VC (*n =* 7)**	**WLC (*n =* 9)**	**Overall (*n =* 34)**
**Caregiver**					
Age (yr)	38.22 ± 2.64	39.56 ± 4.48	37.86 ± 2.80	40.22 ± 3.8	39.03 ± 3.52
**Gender (%)**					
Male	0 (0)	3 (33)	1 (14)	0 (0)	4 (12)
Female	9 (100)	6 (67)	6 (86)	9 (100)	30 (88)
**Education (%)**					
Secondary	1 (11)	4 (44)	1 (14)	3 (33)	9 (26)
Post-secondary	1 (11)	1 (11)	0 (0)	2 (22)	4 (12)
Tertiary or above	7 (77)	4 (44)	6 (86)	4 (44)	21 (62)
**Employment (%)**					
Full-time	4 (44)	5 (56)	6 (86)	8 (89)	23 (68)
Part-time	1 (11)	2 (22)	1 (14)	0 (0)	4 (12)
Unemployed	4 (11)	2 (22)	0 (0)	1 (11)	7 (20)
**Caregiver (%)**					
As the primary	2 (22)	1 (11)	0 (0)	0 (0)	3 (9)
One of the 2 main	2 (22)	5 (56)	0 (0)	1 (11)	8 (23)
Many caregivers	5 (56)	3 (33)	5 (71)	8 (89)	21 (62)
Other (babysitters)	0 (0)	0 (0)	2 (29)	0 (0)	2 (6)
**Child**					
Age (yr)	5.33 ± 1	4.22 ± 1.3	3.71 ± 1.5	4.22 ± 1.48	4.41 +/− 1.40
**Gender (%)**					
Male	6 (67)	6 (67)	7 (100)	9 (100)	28 (82)
Female	3 (33)	3 (33)	0 (0)	0 (0)	6 (18)
With sibling(s) (%)	5 (56)	4 (44)	3 (43)	2 (22)	14 (41)
Sibling(s) with ASD/DD	4 (80)	1 (25)	0 (0)	0 (0)	5 (36)

There were no significant differences in demographic attributes and baseline measurements comparison between those who dropped out and those who completed the study. Therefore, there were no obvious biases attributable to attrition.

### Quantitative outcomes

#### Acceptability

##### Post-session and Home Visit Feedback Form by caregivers

Six questions in the Post-session (PS) Feedback Form-Caregiver and three questions in Home Visit (HV) Caregiver Feedback Form were selected to assess the comprehensiveness and relevance, and agreement with their personal values, duration, and usefulness. The results of comprehensiveness and relevance, and agreement with their personal values were summarized in Table 2, and the results for duration and usefulness were summarized in Table 3.

**Table 2:** Post-session Feedback Form-Caregiver (Comprehensiveness & relevance, agreement with personal values).

	**Comprehensiveness & relevance**			**Conflicts with personal values**		
	***#*** **(Rate of dissatisfaction)**			***##*** **(Rate of confliction)**		
	**EL (*n =* 9)**	**IP (*n =* 9)**	**VC (*n =* 7)**	**EL (*n =* 9)**	**IP (*n =* 9)**	**VC (*n =* 7)**
S1A	0 (0%)	0 (0%)	0 (0%)	0 (0%)	4 (22%)	0 (0%)
S1B	1 (3%)	0 (0%)	0 (0%)	0 (0%)	0 (0%)	0 (0%)
S2	1 (3%)	0 (0%)	0 (0%)	0 (0%)	2 (11%)	2 (14%)
S3	0 (0%)	0 (0%)	0 (0%)	0 (0%)	4 (22%)	0 (0%)
S4	0 (0%)	0 (0%)	0 (0%)	0 (0%)	6 (33%)	0 (0%)
S5	0 (0%)	0 (0%)	0 (0%)	1 (6%)	2 (11%)	2 (14%)
S6	0 (0%)	0 (0%)	0 (0%)	2 (11%)	4 (22%)	3 (21%)
S7	0 (0%)	0 (0%)	0 (0%)	0 (0%)	4 (22%)	2 (14%)
S8	0 (0%)	0 (0%)	0 (0%)	0 (0%)	4 (22%)	2 (14%)

# Value < 3 “Disagree, Strongly disagree.”

## Value > 3 “Agree, Strongly agree.”

**Table 3:** Post-session and Home Visit Feedback Form by Caregivers (Duration, usefulness of home visits and skill coaching).

**Rate of dissatisfaction**
		**S1A**	**S1B**	**S2**	**S3**	**S4**	**S5**	**S6**	**S7**	**S8**	**HV1**	**HV2**	**HV3**
^*^Duration	EL (*n =* 9)	11%	11%	11%	11%	11%	0%	0%	11%	0%	22%	11%	11%
	IP (*n =* 9)	33%	7%	22%	0%	11%	19%	22%	0%	0%	11%	0%	0%
	VC (*n =* 7)	38%	14%	10%	5%	10%	14%	19%	10%	0%	14%	0%	0%
^**^Home visits	EL (*n =* 9)										22%	22%	11%
	IP (*n =* 9)	N/A	N/A	N/A	N/A	N/A	N/A	N/A	N/A	N/A	0%	0%	0%
	VC (*n =* 7)										0%	0%	0%
^**^Skill coaching	EL (*n =* 9)										22%	22%	22%
	IP (*n =* 9)	N/A	N/A	N/A	N/A	N/A	N/A	N/A	N/A	N/A	11%	0%	0%
	VC (*n =* 7)	N/A	N/A	N/A	N/A	N/A	N/A	N/A	N/A	N/A	0%	0%	0%

* Too long / too short.

** Value < 3 “Not very useful, Completely useless.”

Results from the four questions in the Post-session (PS) Feedback Form-Caregiver indicated that except for one of the participants' ratings for sessions 1B and 2 in EL, all participants agreed with the comprehensiveness and relevance of the session contents across all groups.

Regarding the perceived conflicts with contents and their beliefs, IP & VC groups found the contents of sessions conflicting with their personal values, whereas the majority of the EL participants found the contents of sessions neither conflicted with their own or their families' values.

Since there was no session provided to WLC participants, comments on the duration of each session were collected only for EL, IP, and VC groups only, while all groups gave ratings on that of each home visit. Despite a few participants in both groups finding the first session too long, most participants found the duration of other sessions and home visits appropriate.

Participants were asked to comment on the usefulness of home visits and skill coaching. Caregivers, except for one EL, found the first home visit not very useful, and none of the other participants in IP and VC groups found the home visits and skill coaching not useful. On the contrary, some caregivers in EL and WLC groups rated the home visits and skill coaching as “not very useful” or “completely useless.”

##### Facilitator's Feedback Form

Table 4 summarizes the average ratings of the nine sessions by facilitators over IP and VC groups regarding the content of the sessions, the participant's level of engagement, acceptance, and involvement in planning home practices after each session. Only IP and VC groups were facilitated, so only these two groups of data were collected *via* this measure.

**Table 4:** Facilitator's Feedback Form (Contents of the sessions).

	**IP**	**VC**
Caregivers' degree of recognition of the concept of the program	4.7	4.8
Caregivers' sense of engagement and participation	4.8	4.9
Sessions' contents for perceived relevance to caregivers	4.8	4.7
Caregivers' acceptance of contents.	4.7	4.8

The average rating of the four questions in both groups was higher than 4 (out of five), suggesting that caregivers were perceived to have a great recognition of the concept of the programme and a high level of engagement and participation. As for the perceived relevance and acceptance, the results showed inconceivably high too.

#### Feasibility

Feasibility of CST conducted through different delivery modes was evaluated by participants' completion rate, attendance, adherence to home practice, observer's feedback on the involvement of the participants, and implementation of the group sessions.

##### Attendance and completion rate

Both IP and VC groups achieved a high attendance (95% for IP and 100% for VC), which was recorded by facilitators during each session. Coupling with high attendance, the completion rate of these two groups was also high (100% for IP and 78% for VC) which were shown in Table 5. On the contrary, the low attendance of EL group resulted in the lowest completion rate (64%), suggesting that the asynchronous mode favors participants with more self-discipline to learn regularly and complete the programme.

**Table 5:** Attendance of EL, IP & VC groups in each session and completion rate of all groups.

	**Attendance**										**Completion rate**		
	**S1A**	**S1B**	**S2**	**S3**	**S4**	**S5**	**S6**	**S7**	**S8**	**Overall (%)**	**Start**	**End**	**%**
EL (*n* = 9)	9	8	4	6	6	7	8	6	4	72%	14	9	64%
IP (*n* = 9)	9	8	9	8	9	8	8	9	9	95%	9	9	100%
VC (*n* = 7)	7	7	7	7	7	7	7	7	7	100%	9	7	78%
WLC	/	/	/	/	/	/	/	/	/	/	11	9	82%

##### Adherence to home practice

Caregivers' adherence to home practice was evaluated by the feedback in the Caregiver Diary on the frequency and the daily time the participants practiced “Skills and Strategy” in daily activities at T1 & T2.

Table 6 shows the average practice frequency and duration per week for each group. The average practice frequency practiced with the children per week for EL, IP, and VC groups were 3.1 (SD = 4.2), 5.1 (SD = 4.0), and 7.4 (SD = 6.2) at T1, and were 3.2 (SD = 4.1), 6.9 (SD = 5.5), and 4.6 (SD = 4.5) at T2, respectively.

**Table 6:** Comparison of practice frequency and duration.

		**EL (*****n*** = **9)**		**IP (*****n*** = **9)**		**VC (*****n*** = **7)**	
		**Mean**	**SD**	**Mean**	**SD**	**Mean**	**SD**
*^a^*Frequency (Times/wk)	T1	3.1	4.2	5.1	4.0	7.4	6.2
	T2	3.2	4.1	6.9	5.5	4.6	4.5
	*Difference*	*0.1 (3%)*	*0.8*	*1.8 (35%)*	*4.6*	*−2.8 (38%)*	*4.1*
							
*^b^*Duration (Minutes/wk)	T1	50.6	67.1	78.6	94.4	139.3	137.1
	T2	47.0	63.7	83.8	91.3	65.6	56.9
	*Difference*	*−3.6 (−7%)*	*23.3*	*5.2 (7%)*	*27.5*	*−73.7 (−53%)*	*96.6*

a Takes 0.5 time per week for reporting less than one time a week, takes 3.5 times per week for reporting 3-4 times a week, takes 5.5 times per week for reporting 5-6 times a week, takes 2 times a day, i.e., 14 times per week for reporting more than one time a day.

b Takes 22.5 minutes each time for reporting 15-30 min each time, takes 30 min each time for reporting more than 30 min each time.

The average minutes practiced with the children per week for EL, IP, and VC groups were 50.6 (SD = 67.1), 78.6 (SD = 94.4), and 139.3 (SD = 137.1) at T1, and were 47 (SD = 63.7), 83.8 (SD = 91.3), and 65.6 (SD = 56.9) at T2, respectively.

The duration of practice time per week dropped for both EL and VC groups. Although the percentage dropped in both practice frequency and duration per week for the VC group was substantially large (-38% and −53% respectively), the change was not significant due to widespread use of the data. This dramatic drop in VC was mainly because two caregivers reduced the practices from twice a day (14 times per week) to 5–6 times a week. However, it was found that there was an increase in both practice frequency and duration per week in the IP group. Figures also show that both the practice frequency and duration were lower in EL than that in IP and VC groups.

##### Intervention fidelity and feasibility of group sessions delivery

Group sessions' intervention fidelity was reported by observers in rating the participants' degree of comfort, enthusiasm/interest, and level of involvement in planning home practice. Table 7 summarizes the average ratings of the nine sessions on the intervention fidelity of the group sessions. The average rating of the four components in both groups was >4 (out of five), suggesting that the intervention fidelity of group sessions was relatively high for both delivery modes.

**Table 7:** Average ratings on the intervention fidelity and feasibility of group sessions' delivery.

	**Components**	**IP**	**VC**
Intervention fidelity	Caregivers' degree of comfort	4.7	4.5
	Caregivers' enthusiasm/interest	4.6	4.6
	Caregivers' level of confidence	4.5	4.4
	Review of home practice	4.4	4.4
Feasibility	^*^Complexity of the sessions' contents and concepts	3.3	3.1
	Appropriateness of the amount of the sessions	3.4	3.3
	Facilitators' perceived preparedness for the sessions	4.8	4.8

* Value = 3 “Appropriate”; < 3 “A bit simple, too simple”; > 3 A bit complex, too complex.

Complexity, amount of content, and perceived preparation for each session were rated by the teaching facilitator to evaluate the implementation of the programme. Ratings of the complexity of the sessions' contents and concepts, as well as the number of sessions in both groups, were approximate 3 (out of five), indicating that facilitators found these two components of most sessions appropriate. Regarding the perceived readiness for the sessions, facilitators reported very high ratings at 4.8 (out of five) as shown in Table 7, indicating that facilitators were always well-prepared for the sessions.

#### Quality of Life as parent outcome

One-way ANOVA test revealed that there was no significant difference between groups at T0, F < 1. A two-way ANOVA was conducted with a within-participant factor of time points (2 levels: T0, T2) and a between-participant factor of condition (4 levels: EL, IP, VC, WLC). The main effects of both time points and condition were not significant, F_(1,30)_ = 2.55, *p* = 0.12 and *F* < 1. The interaction between time points and conditions was also not significant, *F* < 1.

Descriptive analyses showed that there was an overall improvement in EL, IP and VC groups (3, 13, and 16%, respectively), while WLC slightly worsened (−3%) from T0 to T2. The overall and individual item scores of the GHQ-12 in each group at T0, T2, and the differences between T0 and T2 are shown in Table 8.

**Table 8:** GHQ-12 item and SDQ scores at T0 and T2.

		**EL (*n* = 9)**	**IP (*n* = 9)**	**VC (*n* = 7)**	**WLC (*n* = 9)**
*^a^*GHQ-12	T0	17.33	15.56	13.99	13.45
	T2	16.77	13.57	11.71	13.90
	Diff	*−0.56 (−3%)*	*−1.99 (−13%)*	*−2.28 (−16%)*	*0.45 (3%)*
*^a^*SDQ- Difficulties-total	T0	21.33	21.56	15.72	18.00
	T2	20.00	18.78	13.29	18.00
	Diff	*−1.33 (−6%)*	*−2.78 (−13%)*	*−2.43^#^ (−15%)*	*0 (0%)*
*^b^*SDQ- Prosocial	T0	3.78	2.44	2.00	4.56
	T2	4.00	3.33	2.71	4.67
	Diff	*0.22 (5.9%)*	*0.89^#^ (36.5%)*	*0.71 (35.5%)*	*0.11 (2.4%)*

a Differences between T2 & T0: -ve - improved, +ve – worsened.

b Differences between T2 & T0: +ve - improved, -ve – worsened.

^*^p < 0.05;

# Marginal 0.05 < p < 0.1.

At T2, there were 7 items improved in IP (with Q11 on self-worth yielding a significant change at p <0.05), 6 items in both EL and VC groups, and 3 items in WLC. Whilst, one item worsened in IP, 2 in VC, 3 in EL, and 5 in WLC. Improvement in the IP condition was strongly evidenced as 6 out of these 7 items were with rating below one, meaning IP participants nearly always have positive feelings about those items. One item related to negative feelings, meaning they rarely or do not feel that way at all.

#### Strengths and Difficulties Questionnaire as child outcome

A two-way ANOVA was conducted for *SDQ total* (Table 8) with a within-participant factor of time points (2 levels: T0, T2) and a between-participant factor of condition (4 levels: EL, IP, VC, WLC), the main effect of time points was significant, with F_(1, 30)_ = 5.55, *p* < 0.05, partial eta squared = 0.16. The main effect of conditions was also significant, F_(3, 30)_ = 3.21, *p* < 0.05, partial eta squared = 0.24. The interaction between time points and condition was not significant, *F* < 1. Further investigation showed that the SDQ total of IP was significantly lower at T2 than at T0 (*p* < 0.05) and VC was significantly lower than that of EL at T2 (*p* < 0.05).

A two-way ANOVA was conducted for prosocial with a within-participant factor of time points (2 levels: T0, T2) and a between-participant factor of condition (4 levels: EL, IP, VC, WLC), the main effects of both time points [F_(1, 30)_ = 3.65, *p* = 0.07, partial eta squared = 0.11] and condition [F_(3, 30)_ = 1.54, *p* = 0.23, partial eta squared = 0.13] were not significant. The interaction between time points and condition was also not significant, *F* < 1.

The average scores of the Difficulties-total for EL, IP, VC, and WLC were 21.33 (SD = 5.39), 21.56 (SD = 4.64), 15.72 (SD = 3.64), and 18.00 (SD = 5.48) at T0, and were 20.00 (SD = 5.61), 18.78 (SD = 4.41), 13.29 (SD = 2.56), and 18.00 (SD = 5.05) at T2, respectively.

The average scores of the Prosocial Scale for EL, IP, VC, and WLC were 3.78 (SD = 2.11), 2.44 (SD = 2.65), 2.00 (SD = 2.16), and 4.56 (SD = 2.60) at T0, and were 4.00 (SD = 1.94), 3.33 (SD = 3.16), 2.71 (SD = 1.25), and 4.67 (SD = 2.83) at T2, respectively.

Descriptive analyses showed that there was an overall improvement (decrease in problematic behaviors and increase in prosocial behaviors) in all experimental groups, while the total difficulties score of the WLC group remained unchanged. In particular, the two experimental groups with facilitators (IP and VC) gained greater improvements (with 13% and 15% decrease in Difficulties-total, and 36.5% and 35.5% increase in Prosocial Scale for IP and VC groups, respectively) than the group without a facilitator (EL group), with only 6% decrease in Difficulties-total and 5.9% increase in Prosocial Scale. This suggests that the presence of facilitators can be a factor in the decrease in problematic behaviors and increase in prosocial behaviors among children.

### Qualitative outcomes

Open-ended feedback on each session and each home visit that was received across the groups underwent pattern coding and major themes were established. Three underlying themes including “Delivery Format,” “Programme Materials” and “Interaction” were identified and further broken down into various sub-themes under the domain of acceptability and feasibility with both positive and negative valence. Table 9 shows the descriptions of the comments reported vastly in the four groups, in which heavyweight subthemes are described qualitatively hereafter and noteworthy subthemes are evidenced with group identifiers. As the video recording arrangement is a standalone question in the Post Home Visit Feedback Form, the finding is separately described in a distinct section below.

**Table 9:** Themes derived from comments of each Post-session & Home Visit.

	***Theme***	***Valence***	***Subthemes grouped over feedbacks on sessions and home visits***	**EL**	**IP**	**VC**
**Acceptability**	***Delivery format***	+ve	Perceived convenience (PS/HV)	✓	✓	✓
			Elevated privacy (PS/HV)	✓	✓	✓
			Support to video-recording (HV)	✓	✓	✓
		-ve	Barriers to video-recordings (HV)	✓	✓	✓
	***Programme materials***	+ve	Comprehensible content (PS)	✓	✓	✓
	***Interaction***	+ve	Supportive peers' sharing (PS)		✓	✓
			Interactive professional facilitation on skill application and examples (PS)	✓	✓	✓
			Instant professional coaching at home visits (HV)	✓	✓	✓
		-ve	No interactions with peers (PS)	✓	✓	✓
			Limited professional guidance or feedback (PS/HV)	✓	✓	✓
**Feasibility**	***Delivery format***	+ve	Effective use of multimedia materials (PS)	✓	✓	✓
		-ve	Time control (PS)	✓	✓	✓
			Large number of participants (PS)			✓
			Challenges to complete the sessions without a fixed time session schedule (PS/HV)	✓	✓	✓
			Videos taken being too lengthy (HV)	✓	✓	✓
	***Programme materials***	+ve	Effective skills & strategies (PS/HV)	✓	✓	✓
		-ve	Insufficient content or illustration of examples in some of the sessions (PS)	✓	✓	✓
	***Interaction***	+ve	Face-to-face skill practices (PS/HV)		✓	
			In-person demonstration with children (HV)	✓	✓	✓
		-ve	Lack of in-person skill practices/in-person home visits (PS/HV)	✓	✓	✓

Findings show that there was a high level of acceptability and feasibility of the adapted programme in the aspects of delivery formats and programme materials. As for interaction, it was the most popular theme among IP and VC participants while it became the deficient factor to be accepted and implemented in EL and WLC groups where interactions were limited.

#### Delivery formats

Perceived convenience and increased privacy raised the acceptability, and effective use of multimedia boosted the feasibility of the adapted delivery formats. Convenience was viewed differently in both group sessions and home visits by different groups. EL enjoyed the time and location flexibility, as well as the room to learn at their own pace. Some revealed that the self-learning modes could fit the busy working parents while privacy could be preserved.

EL11: “The self-learning sessions were comprehensive and suited busy working mothers.”

Group sessions were considered to be convenient among IP & VC as they strictly followed the social distancing measures where in-person facilitation was not allowed. Besides, IP participants also appreciated the privacy of one-on-one home visits too.

IP01: “The advantage of doing it via recording is that it is less intrusive. The family and the child may not notice strangers around and therefore help create a more home-like environment.”

Nevertheless, poor time management and a large number of participants in the groups lowered the feasibility of IP and VC. Unstructured learning decreased the feasibility of EL. Whilst barriers to taking videos and length of videos were the negative factors in implementing the programme across all groups.

#### Programme materials

Comprehensive session content and efficacious skill and strategies were the acceptable and feasible elements of the programme irrespective of the intervention modes.

EL12: “This session (session 2 on joint engagement) changed my mindset. It made me understand that I was too instructive while playing with my kid, and from now on, I have to prepare two sets of toys to imitate his way of playing.”

However, some responses showed that there were insufficient contents or examples in the materials, for sessions 5, 6, and 7 (behavioral management and teaching skills). This can be improved accordingly to increase the feasibility of the programme materials.

EL05: “How to deal with self-harming behaviors?”

VC11: “This topic is very complicated, and I recommend having in-depth discussions, for example, through the case study.”

#### Interactions

Interactions of any kind were deemed as an important factor in acceptability and feasibility across. Subthemes identified were supportive peers' sharing, interactive professional facilitation on skills application with more examples, interactive professional coaching during home visits for the acceptability domain, and face-to-face skill practices and in-person skill demonstrations with children for the feasibility domain. All IP participants reported that they enjoyed and appreciated much with all the listed delivery elements at group sessions and home visits, and thus they showed a high level of acceptability and feasibility. Some of them even gave “perfect” in their comment. Responses revealed there were perceived positive changes in better understanding of children's communication, better handling of children's behaviors, improvement in parent-child relationship, and harmony in the family which led to better family functioning.

IP09: “I learnt at the skill coaching section from [CST interventionist] and the facilitators which were practical and applicable to deal with SEN children. I could better understand my child and better handle her behaviors. There are fewer conflicts in the family and my hubby appreciated a lot of the changes. We no longer argued as much as before and it's magical!”

As for VC, they also had a high level of acceptability as they were offered the interactive elements as IP. They appreciated the professional facilitation at group sessions and the instant professional coaching at home visits online. Yet, they would suggest the addition of face-to-face skill practices and in-person skill demonstrations with children for VC delivery modes. Meanwhile, the majority of EL participants complained about lacking professional guidance or feedback and interactions with peers in addition to face-to-face skill practices and in-person skill demonstrations with children. They were frustrated as they perceived no improvements in their parenting skills. Also, they found the home visits not helpful as no instant feedback was provided. A few participants desperately requested interaction as:

EL07: “I am so lonely and cannot improve my skills without interactions and feedback.”

#### Arrangement of video recordings at the home visits

All participants in three interventions accepted video recording as a necessary part of the first home visit. This acceptability remained across the rest of the two home visits in IP and VC while the acceptability in EL had been dropping since the second home visit. Participants reported that they understood the arrangement as essential and as an alternative under the pandemic situation while a majority understood and expected that the videos taken would receive professional feedback on their practice, serve as a self-learning opportunity to improve their skills and be a part of sustaining the programme in the long run.

EL09: “Both my child and I enjoyed the video-recording very much.”

IP11: “The videos can be a reference and review the child's development in the future.”

VC09: “Routines are important in training the children with SEN. Video taking is crucial in assisting the assessment of their abilities and in developing them.”

Despite the acceptability, some of them complained about the “barriers to video-recordings,” which included the distractions from other family members at home and technical problems of using electronic equipment to capture the videos and uploading the videos to the platform.

## Discussions

This study sought to investigate the acceptability and feasibility of various implementation methods of CST among families with children with developmental disabilities in Hong Kong under lockdown.

High quantitative ratings for comprehensiveness and relevance, in line with their personal values, duration, and usefulness at post-session and post home visit among the participants indicate high acceptance and satisfaction with CST across various delivery modes.

As per the qualitative findings, the IP delivery mode received the highest level of acceptability and feasibility, and VC came second with the satisfied comments received in general. Then, EL followed VC, and WLC showed the lowest acceptance and feasibility. Items with negative valence under IP and VC groups are controllable factors, such as better time management and smaller group size. Furthermore, the suggestions of additional content and examples in behavior management, echoed the caregivers' needs in an Italian study (34), and provided more alternatives to mitigate challenging behaviors such as self-harming behaviors are suggested.

Comments from EL also provided insights and suggestions for delivering the programme with improved acceptability and feasibility. This is aligned with the key elements of interactions - two-way communication - which can be online or in-person, and with facilitators or with peers. Caregivers of children with developmental delays or disabilities in Hong Kong are special caregivers with out-of-proportional parenting stress (16–18). Although they enroll in the programme to acquire knowledge on parenting, they also wish for more guidance, support, encouragement, and recognition. EL caregivers in the study reported being frustrated by the lack of feedback and experiencing loneliness that was not mitigated by the intervention. Besides, World Health Organization's Caregiver Skills Training Programme (WHO CST) is a skill transferring programme that skill practices with interactive feedback would be more beneficial for the skill-attaining process. In addition, the demand for interactive activities increased with the number of sessions and home visits delivered and the demands were more desperate with time.

In view of EL feasibility, the more flexible self-learning mode appears more suitable for caregivers who are less busy and have better time management skills. Caregivers reported that they were not able to complete the sessions within a week because of work commitments, which is reflected in their attendance. Regular weekly reminders were given to those who had not completed the previous session.

With the clinical outcomes, IP and VC yielded greater improvements in SDQ and GHQ-12 than EL, including the well-being of caregivers, perceived children's difficulties, and prosocial behaviors, which are triangulated with the acceptability and feasibility levels of delivery modes. Results of SDQ show an overall improvement (decrease in problematic behaviors and increase in prosocial behaviors) in all experimental groups, while the total difficulties score of the WLC group remained unchanged. In particular, the two experimental groups with facilitators (IP and VC) gained greater improvements (with 13% and 15% decrease in Difficulties-total, and 36.5% and 35.5% increase in Prosocial Scale for IP and VC groups, respectively) than the group without a facilitator (EL group), with only 6% decrease in Difficulties-total and 5.8% increase in Prosocial Scale. This suggests that the presence of facilitators can be a factor in the development of caregiver skills for decreasing challenging behaviors and increasing prosocial behaviors.

From GHQ-12 results, all groups involved in CST showed positive results, while the group (EL) without a facilitator showed improvement, but to a lesser extent. A decreased GHQ-12 score in the control group participants may be due to the challenges and frustrations in engaging their children during the video recording. These results also echoed qualitative findings that the programme helped improve caregivers' well-being, child's communication and behaviors, and thus better family functioning. Concerning the value conflict, the quantitative result was mixed. Thereafter, further analysis revealed that individual participants showing a conflict in personal values did have positive changes in the GHQ-12 results. This suggests that conflicting information does not necessarily obstruct the caregivers from learning new concepts. The intervention may prompt caregivers to revisit their prior beliefs that result in positive improvements in their mental health and quality of life.

In other words, participants perceived both in-person and videoconferencing modalities favor skill attainment among parents strengthening the communication and behavioral management with their children. These were supported by the results of GHQ and SDQ that VC and IP promoted caregivers' well-being and child's adaptive behaviors and reduced challenging behaviors among the children. All groups, except the wait-list control group, showed some positive changes in SDQ, which suggest the video recordings as data collection may increase their awareness of the interaction with their child, resulting in certain improvements. Overall, only IP gained significant improvements while other groups did not show any advantage. This indicates the in-person live practice sessions, and skill coaching during the home visits played a role in the improvement of children's behaviors.

### Limitations

There were several limitations to this study. First, the initial dropout rate (51%) resulted in a dramatic decrease in sample size from 70 to 34 due to the sudden pandemic spike. Some participants became more concerned about social distancing in face-to-face mode while others who expected in-person sessions dropped out when they were assigned to non-interacting modes. This reduced the power to detect differences between the groups, in particular, IP and VC, but not EL groups. A more accurate understanding of the effect of the experimental conditions could only be obtained when the study is conducted under normal circumstances. The current results however would be informative to the understanding of the experimental effect under a pandemic situation with disrupted social interaction. Second, the outbreak of COVID-19 disrupted the original research for the IP group, leading to swapping the face-to-face sessions to online sessions, like VC, which lowered the difference between the two groups. It might have affected the research outcomes. Lastly, although the results promote confidence in further expansion of implementation, the study has less experimental control over confounding factors, which may affect the persuasiveness of the results.

All in all, this study showed supportive evidence of the acceptability and feasibility of the World Health Organization's Caregiver Skills Training Programme (WHO CST) conducted by different delivery modes, including in-person, virtual, and self-learning, among caregivers of children with developmental disabilities. This study only reported two measures related to caregivers' quality of life and children's difficulties and adaptive behaviors, so future studies are needed to examine other related variables, such as caregivers' coping skills, joint engagement, and children's behaviors in different domains with larger sample size. To address comments from caregivers, future research on effectiveness can be carried out with further adaptations to the current delivery modes with more interactive elements incorporated.

## Conclusions

Current findings indicate that three adapted CST delivery modes were found highly acceptable and feasible. Participants indicated that the programme was comprehensive, reliable, and useful despite the different ways the programme was delivered. To conclude, the programme was determined to have high acceptability and feasibility, regardless of the mode of delivery. When comparing the degree of effectiveness, all three modes of delivery yielded positive impacts on caregivers and children. In-person delivery mode was found to have the greatest improvement in mitigating perceived child's challenging behaviors, whereas the interactive modes, IP and VC, showed the larger positive change in improving caregivers' well-being, which was followed by EL mode. In-person skill practices can significantly improve children's behaviors whereas personal support, either face-to-face or virtual, appears to be key in maintaining caregivers' mental well-being. In addition, caregivers highlighted that having facilitators' face-to-face coaching and interactive peer support contributed to their persistence and momentum to complete the programme. The findings can be linked to how the programme nature can be highlighted to satisfy the objectives of the programme.

On top of this, the heterogeneous findings suggest that it is difficult to conclude which service delivery mode, eLearning, videoconferencing, or in-person, is better. Self-directed programmes usually provide a convenient means for users to learn at their own pace, without being restricted by time, location, or other tangible conditions (82). It appears to be most suited to caregivers who have strong independent learning and self-management skills. However, for some intervention programmes based on a more complex theoretical design, self-learning alone may not give desirable outcomes. On the other hand, programmes requiring a high degree of interaction to demonstrate ways to teach new skills and manage behaviors, or those interventions requiring meticulous observations of subtle expressions or body language, and immediate reciprocity in psychotherapy sessions may not have a good fit with the existing technological level. Under such circumstances, the traditional face-to-face mode still has advantages (83). Furthermore, service receivers are different populations, so the acceptance and capability of technology applications can vary considerably (84), which goes against a “one-size-fits-all” approach. Offering caregiver-mediated interventions in a variety of delivery modes may best meet the needs of a diverse group of caregivers and maximize the reduction in the treatment gap for children with developmental disabilities and delays and their families. Therefore, future research should be focused on the advantages of each service delivery mode and how they can fit into the existing community settings and fit the target beneficiaries. It is also necessary to investigate the factors that affect the efficacy of the intervention, so as to exclude the possibility of erroneously attributing the results of the intervention to the service delivery modes.

Different CST modes of delivery were found acceptable and feasible whereas the statistical GHQ-12 and SDQ findings gave different degrees of positive changes among the delivery modes. In this study, the in-person mode received the highest acceptability and feasibility, and the most desirable clinical outcomes. Findings also can be interpreted that the degree of interaction was a positive factor in its effectiveness. Various interactive facilitation components can be considered and incorporated to fit best into the needs of the caregivers as well as the practitioners. This serves as a direction for how World Health Organization's Caregiver Skills Training Programme (WHO CST) can be implemented in Hong Kong, or a reference to other countries implementing the programme in their community, when balancing the effectiveness and constraints in different settings.

## Data availability statement

The raw data supporting the conclusions of this article will be made available by the authors, without undue reservation.

## Ethics statement

The studies involving human participants were reviewed and approved by the Human Research Ethics Committee of Hong Kong University (EA200178). All data collection was performed in accordance with HKU guidelines and regulations. The patients/participants provided their written informed consent to participate in this study.

## WHO CST team

Chiara Servili^1^, Erica Salomone^1,2^, Laura Pacione^1,3^, Stephanie Shire^4^ and Felicity L. Brown^5,6^

^1^Department of Mental Health and Substance Use, World Health Organization, Geneva, Switzerland
^2^The University of Milano-Bicocca, Milan, Italy
^3^Department of Psychiatry, Division of Child and Youth Mental Health, University of Toronto, Toronto, ON, Canada
^4^The University of Oregon, Eugene, OR, United States
^5^Research and Development Department, War Child Holland, Amsterdam, Netherlands
^6^Amsterdam Institute of Social Science Research, University of Amsterdam, Amsterdam, Netherlands.

## Author contributions

Material preparation, data collection and analysis were performed by JL, SL, and FI. The first draft of the manuscript was written by JL and FI. All authors contributed to the study conception and design, commented on previous versions of the manuscript, and read and approved the final manuscript.

## Conflict of interest

The authors declare that the research was conducted in the absence of any commercial or financial relationships that could be construed as a potential conflict of interest.

## Publisher's note

All claims expressed in this article are solely those of the authors and do not necessarily represent those of their affiliated organizations, or those of the publisher, the editors and the reviewers. Any product that may be evaluated in this article, or claim that may be made by its manufacturer, is not guaranteed or endorsed by the publisher.
