# Supplementary material for: Acceptability and feasibility of the World Health Organization's Caregiver Skills Training Programme (WHO CST) delivered via eLearning, videoconferencing, and in-person hybrid modalities in Hong Kong
Source: Front Psychiatry. 2022 Sep 7;13:915263. doi: 10.3389/fpsyt.2022.915263 (PMC9511500; doi:10.3389/fpsyt.2022.915263)
Supplement: Supplementary file 1 [file Table_1.doc]

Appendix - Summary of the adaptations in EL, IP Hybrid and VC delivery modes

| **Modes* Key Elements** |  | **eLearning** |  | **In-person Hybrid** |  | **Videoconferencing** |  | **Wait-List Control** |
| --- | --- | --- | --- | --- | --- | --- | --- | --- |
| **GROUP SESSION** | | | | | | | | |
| **Platform &  Session Format** |  | Via encrypted online platform,  which can only be accessed  with the registered account. Platform session links are disseminated via email at a fixed time on a weekly basis. |  | Nine sessions of 2.5-3 hours long are delivered by two facilitators via Zoom & at a weekly fixed time, in which one hour of the 3 sessions (3rd, 6th & 9th) on live skill practices are conducted in-person at facility setting |  | Nine sessions of 2.5-3 hours long are delivered by two facilitators via Zoom & at a weekly fixed time |  | No sessions are given |
| **Wellness Activity** |  | Participants practise wellness activity with a pre-recorded relaxation video with the flow of the session |  | Participants practise wellness activity with a pre-recorded relaxation video via Zoom |  | Participants practise wellness activity with a pre-recorded relaxation video via Zoom |  |
| **Review on  Previous Session** |  | Key messages and strategies are reviewed with notes on the platform |  | Led by facilitators interacting with participants in the session via Zoom |  | Led by facilitators interacting with participants in the session via Zoom |  |
| **Sharing on  Home Practices** |  | Participants jot down their experiences & challenges under the Home Practices Sharing on the platform |  | Every participant is invited to share their experiences and challenges practising with children via Zoom |  | Every participant is invited to share their experiences and challenges practising with children via Zoom |  |
| **Introduction of  Key Messages,  Skills & Strategies** |  | Key messages & strategies are concluded after participants have gone through stories & discussions on the platform |  | Facilitators bring out the key messages, skills and strategies after the illustration through story sharing and thorough discussions via Zoom |  | Facilitators bring out the key messages, skills and strategies after the illustration through story sharing and thorough discussions via Zoom |  |
| **Stories & Discussions** |  | Participants read through the stories & self-reflect on the discussion questions on the platform |  | Stories are either read out by facilitators or among the participants present in the room, and discussions are followed after |  | Stories are either read out by facilitators or among the participants present in the room, and discussions are followed after |  |
| **Role Plays** |  | Role plays are replaced with pre-recorded demonstration videos as scripted in the facilitator guide |  | Role plays are replaced with pre-recorded demonstration videos as scripted in the facilitator guide. In-person role plays are included at three live skill practice sessions |  | Role plays are replaced with pre-recorded demonstration videos as scripted in the facilitator guide |  |
| **Practice in Pairs** |  | Practices with children are suggested in EL |  | Participants are practised the skills & strategies taught in the session with their children on live and instant coaching are given by facilitators via Zoom. Practice in Pairs with another participant are included at three live skill practice sessions |  | Participants are practised the skills & strategies taught in the session with their children on live and instant coaching are given by facilitators via Zoom |  |
| **Plan for Home Practices** |  | Participants jot down their plans for home practices on the platform |  | Participants plan for their home practices which are shared in the sessions via Zoom. In-person sharing on home practice plans are included at three live skill practice sessions |  | Participants plan for their home practices which are shared in the sessions via Zoom |  |
| **HOME VISITS** | | | | | | | | |
| **Rapport Building** |  | Pre-recorded video to greet the family via the platform |  | Greet the family  at the facility setting in-person |  | Greet the family  via Zoom |  | Pre-recorded video to greet the family via the platform |
| **Information collection or review on learnt key messages & strategies** |  | Intake with participants on family & child's background or review of information via the questions on the platform |  | Intake with participants on family & child's background or review of information face-to-face |  | Intake with participants on family & child's background or review of information via Zoom |  | Intake with participants on family & child's background or review of information via the questions on the platform |
| **Parent-child interactions** |  | Two 10-12-min clips on play and home routines under the home settings be submitted via platform |  | Two 10-12-min clips on play and home routines under the home settings be submitted via platform prior to the in-person visit to the facility setting |  | Two 10-12-min clips on play and home routines under the home settings be submitted via platform |  | Two 10-12-min clips on play and home routines under the home settings be submitted via platform |
| **Skill Coaching** |  | No instant coaching are given, while feedbacks are given after the intervention |  | Interact with the child and demonstrate strategies to coach the caregiver on those strategies |  | Comments on the parent-child interactions and caregivers Interact with their children with instant coaching given on those strategies via Zoom |  | No instant coaching are given, while feedbacks are given after the intervention |
| **Goal Setting** |  | Goal setting or revising by the participants which is recorded  at the platform |  | Goal setting or revising with participants face-to-face |  | Goal setting or revising with participants via Zoom |  | Goal setting or revising by the participants which is recorded  at the platform |
| **Plan for Future** |  | Planning for the future on coming to the next session, trouble-shooting and practices in the future by answering the questions at the platform |  | Planning for the future on coming to the next session, trouble-shooting and practices in the future in-person |  | Planning for the future on coming to the next session, trouble-shooting and practices in the future via Zoom |  | Planning for the future on coming to the next session, trouble-shooting and practices in the future by answering the questions at the platform |
